# Paving the road toward the use of β-Fe_2_O_3_ in solar water splitting: Raman identification, phase transformation and strategies for phase stabilization

**DOI:** 10.1093/nsr/nwaa039

**Published:** 2020-03-09

**Authors:** Ningsi Zhang, Xin Wang, Jianyong Feng, Huiting Huang, Yongsheng Guo, Zhaosheng Li, Zhigang Zou

**Affiliations:** Collaborative Innovation Center of Advanced Microstructures, National Laboratory of Solid State Microstructures, College of Engineering and Applied Sciences, Nanjing University, Nanjing 210093, China; Collaborative Innovation Center of Advanced Microstructures, National Laboratory of Solid State Microstructures, College of Engineering and Applied Sciences, Nanjing University, Nanjing 210093, China; Collaborative Innovation Center of Advanced Microstructures, National Laboratory of Solid State Microstructures, College of Engineering and Applied Sciences, Nanjing University, Nanjing 210093, China; Collaborative Innovation Center of Advanced Microstructures, National Laboratory of Solid State Microstructures, College of Engineering and Applied Sciences, Nanjing University, Nanjing 210093, China; Collaborative Innovation Center of Advanced Microstructures, National Laboratory of Solid State Microstructures, College of Engineering and Applied Sciences, Nanjing University, Nanjing 210093, China; Collaborative Innovation Center of Advanced Microstructures, National Laboratory of Solid State Microstructures, College of Engineering and Applied Sciences, Nanjing University, Nanjing 210093, China; Jiangsu Key Laboratory of Nano Technology, Nanjing University, Nanjing 210093, China; Collaborative Innovation Center of Advanced Microstructures, National Laboratory of Solid State Microstructures, College of Engineering and Applied Sciences, Nanjing University, Nanjing 210093, China; Jiangsu Key Laboratory of Nano Technology, Nanjing University, Nanjing 210093, China

**Keywords:** solar energy conversion, metastable phase, phase transformation, iron oxide, photoelectrochemical water splitting

## Abstract

Although β-Fe_2_O_3_ has a high theoretical solar-to-hydrogen efficiency because of its narrow band gap, the study of β-Fe_2_O_3_ photoanodes for water splitting is elusive as a result of their metastable nature. Raman identification of β-Fe_2_O_3_ is theoretically and experimentally investigated in this study for the first time, thus clarifying the debate about its Raman spectrum in the literature. Phase transformation of β-Fe_2_O_3_ to α-Fe_2_O_3_ was found to potentially take place under laser and electron irradiation as well as annealing. Herein, phase transformation of β-Fe_2_O_3_ to α-Fe_2_O_3_ was inhibited by introduction of Zr doping, and β-Fe_2_O_3_ was found to withstand a higher annealing temperature without any phase transformation. The solar water splitting photocurrent of the Zr-doped β-Fe_2_O_3_ photoanode was increased by 500% compared to that of the pure β-Fe_2_O_3_ photoanode. Additionally, Zr-doped β-Fe_2_O_3_ exhibited very good stability during the process of solar water splitting. These results indicate that by improving its thermal stability, metastable β-Fe_2_O_3_ film is a promising photoanode for solar water splitting.

## Teaser

The use of element doping to prevent the phase transition of metastable semiconductors has been approved for the application of photoelectrochemcial hydrogen production via water splitting for the first time.

## INTRODUCTION

Since the concept of a hydrogen economy was introduced, solar hydrogen production using photoelectrochemical (PEC) or photocatalytic water splitting has occupied a very important position in the artificial utilization of inexhaustible solar energy [1–6]. Solar water splitting for hydrogen production has thereby attracted intensive and ever-increasing interest from many researchers [7–15]. The solar-to-hydrogen efficiency of PEC water splitting reached over 19% using III-V multi-junction semiconductors (including In and Ga) prepared by molecular beam epitaxial growth [16]. Unfortunately, there is a lack of low-cost, environmentally friendly, efficient and stable photoelectrodes for PEC water splitting [17–22], thus hindering application of PEC cells for hydrogen production.

In recent decades, iron-based semiconductors with narrow band gaps (e.g. α-Fe_2_O_3_) have been considered as promising photoanode materials with respect to nontoxicity, cost, stability and theoretical solar-to-hydrogen efficiency [23–34]. Recently, a β-Fe_2_O_3_ semiconductor with a direct band gap of 1.9 eV was reported by the present authors as a water splitting photoanode under Air Mass 1.5 Global spectrum (AM 1.5 G, 100 mW cm^–2^) illumination [35]. Theoretically, a β-Fe_2_O_3_ photoanode for solar water splitting may exhibit a solar-to-hydrogen efficiency of 20.9% because of its narrow band gap of 1.9 eV, whereas its solar photocurrent density is still very low [35]. The phase transformation of metastable β-Fe_2_O_3_ to mature α-Fe_2_O_3_ may occur initially at the surface or interface during fabrication of photoanode films because metastable β-Fe_2_O_3_ cannot withstand the high temperature of annealing.

Phase identification of β-Fe_2_O_3_ is very important to avoid the effects of impurities (e.g. α-Fe_2_O_3_) during the process of solar water splitting. Phase characterization of inorganic materials is frequently achieved with such methods as X-ray diffraction (XRD), neutron diffraction, electron diffraction, Raman scattering spectroscopy and infrared absorption spectroscopy. Among these methods, Raman spectroscopy is fast and highly sensitive toward material surface structural information. However, Raman spectra of β-Fe_2_O_3_ (including Raman peak position and shape) reported in the literature are often contradictory [36,37]. Thus, this study aims to clarify the debates surrounding the Raman spectra of β-Fe_2_O_3_.

The wavelength and power of the excitation laser should be chosen carefully during the process of Raman detection. An appropriate wavelength of laser should be selected to enhance the Raman sensibility and minimize the fluorescence emission of the samples. It is equally important that the power of the laser be examined cautiously to avoid the influences of thermal effects caused by the laser, especially for those materials with poor thermal stability. For example, phase transformation of ϵ-Fe_2_O_3_ and γ-Fe_2_O_3_ may take place under laser irradiation [38,39], and β-Fe_2_O_3_ may be converted into α-Fe_2_O_3_ upon heat treatment [35,40]. Therefore, low-power laser irradiation is required to avoid phase transformation of the β-Fe_2_O_3_ samples.

In this study, the influence of laser wavelength and irradiation power on phase transformation of β-Fe_2_O_3_ to α-Fe_2_O_3_ was investigated, and the Raman vibrational spectrum of β-Fe_2_O_3_ was clarified. After being doped with Zr, the particle-assembled β-Fe_2_O_3_ photoanodes can withstand higher annealing temperatures during the post-treatment process. Therefore, the solar water splitting photocurrent density for the particle-assembled Zr-doped β-Fe_2_O_3_ photoanodes was improved significantly to 1.2 mA cm^–2^, which is five times greater than that of pure β-Fe_2_O_3_ photoanodes. Thus, this study suggests metastable β-Fe_2_O_3_ films as promising photoanode materials for solar water splitting.

## RESULTS AND DISCUSSION

β-Fe_2_O_3_ belongs to the space group ${\rm{Ia}}\bar{3}$, T_h_^7^. Irreducible representations of the Γ-point phonon modes for β-Fe_2_O_3_ are shown in Supplementary Table S1. Factor group analysis of β-Fe_2_O_3_ predicts 49 phonon modes, 22 and 17 of which are Raman active modes and infrared active modes (Supplementary Table S2), respectively. Some Raman vibrational modes of β-Fe_2_O_3_ were observed in previous studies, whereas Rahman and coworkers reported different Raman spectra for β-Fe_2_O_3_ [36,37]. To clarify the Raman vibrational modes of β-Fe_2_O_3_, the Raman scatter spectrum of β-Fe_2_O_3_ was verified, as shown in Supplementary Fig. S1. Twelve clear vibrational peaks of β-Fe_2_O_3_ were observed at 158, 169, 234, 258, 274, 314, 328, 368, 386, 397, 522 and 635 cm^–1^ using the 785 nm laser of 0.4 W. These peaks can clearly be distinguished from the Raman vibration peaks of α-Fe_2_O_3_ (227, 246, 293, 300, 411, 499, and 613 cm^–1^). Compared to previous results from Liang and van de Krol [36], four new vibrational peaks at 258, 397, 522 and 635 cm^–1^ were observed, whereas the vibrational peak at 208 cm^–1^ was not observed.

The theoretical calculations and experimental values of Raman vibrational peaks are listed in Supplementary Table S3. Among the 22 vibrational modes predicted by the theoretical calculations, only 12 significant peaks were observed in the experiments. Three weak vibrational modes are marked with asterisks. The M10 (383 cm^–1^) and M11 (389 cm^–1^) modes merge into one peak (386 cm^–1^) in the experimental spectrum. As given in Supplementary Fig. S1, typical peaks corresponding to vibrational modes 158 cm^–1^ (T_g_), 274 cm^–1^ (T_g_), 368 cm^–1^ (T_g_) and 386 cm^–1^ (E_g_+T_g_) can be used as a characteristic spectrum for identifying the phase of β-Fe_2_O_3_. In addition, the theoretically calculated infrared vibrational peaks and the experimental infrared absorption spectrum are also given in Supplementary Table S4 and Supplementary Fig. S2, respectively.

**Figure 1.**
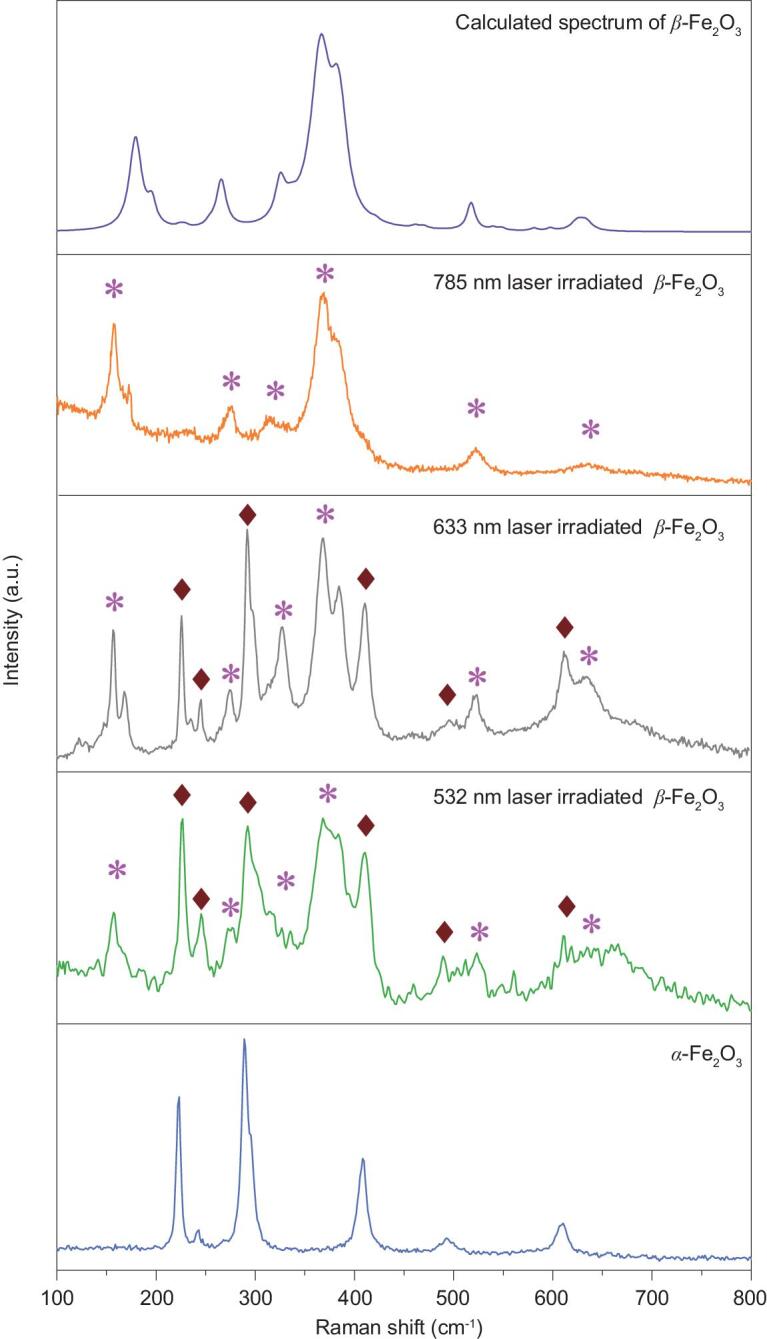
Theoretical calculation and experimental measurement of Raman spectra of β-Fe_2_O_3_ samples excited by a 0.4 mW laser at 785, 633 and 532 nm. The Raman spectrum of α-Fe_2_O_3_ is listed as a reference.

The metastable β-Fe_2_O_3_ phase may be converted to the mature α-Fe_2_O_3_ phase upon laser irradiation of different wavelengths and power. Figure 1 shows the effects of laser wavelength on the Raman spectrum of β-Fe_2_O_3_. The Raman peaks at 227, 293, 411, 499 and 613 cm^–1^ belonging to α-Fe_2_O_3_ appeared after irradiation of the β-Fe_2_O_3_ samples with a 532 nm or 633 nm laser. In the case of 785 nm laser irradiation, the experimental and theoretical Raman peaks for β-Fe_2_O_3_ match in shape but exhibit a slight shift. These results suggest that a 785 nm laser is ideal for detecting the Raman spectra of metastable β-Fe_2_O_3_.

**Figure 2.**
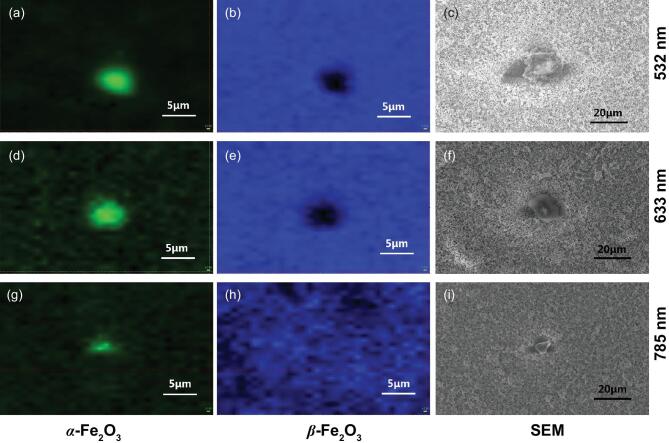
Raman mapping and SEM images of particle-assembled β-Fe_2_O_3_ films excited by a 0.4 mW laser at 785 nm after irradiation by a 10 mW laser with various wavelengths: (a-c) 532 nm, (d-f) 633 nm and (g-i) 785 nm. (a, d, g) α-Fe_2_O_3_; (b, e, h) β-Fe_2_O_3_.

Raman spectra mapping images shown in Fig. 2 also indicate phase transformation of the particle-assembled β-Fe_2_O_3_ films without any necking treatment, in which the α-Fe_2_O_3_ phase appears when the β-Fe_2_O_3_ film samples are irradiated with a 10 mW laser of 532 and 633 nm. Scanning electron microscope (SEM) images also suggest this phase transformation. Even when β-Fe_2_O_3_ was irradiated with a laser of 785 nm with 10 mW power, slight damage of the β-Fe_2_O_3_ films was observed.

**Figure 3.**
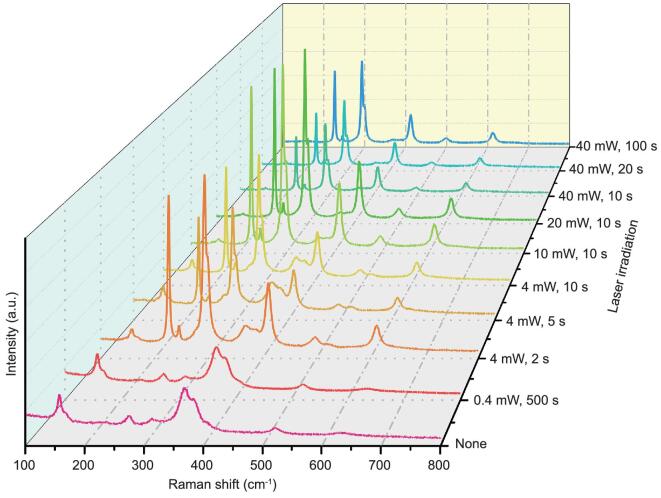
Raman spectra of β-Fe_2_O_3_ under different power laser irradiations of 785 nm.

Additionally, the effects of laser power on the Raman spectrum of β-Fe_2_O_3_ were investigated. When the power of the 785 nm laser is increased, a phase change of β-Fe_2_O_3_ to α-Fe_2_O_3_ possibly occurs (Fig. 3). After β-Fe_2_O_3_ is irradiated by 0.4 mW 785 nm laser for an extended period of 500 s, there is no signal corresponding to α-Fe_2_O_3_. A significant phase transformation occurs with as little as 2 s irradiation with a 4 mW 785 nm laser. As the laser power increases and the exposure time is prolonged, all the β-Fe_2_O_3_ gradually converts to α-Fe_2_O_3_.

**Figure 4.**
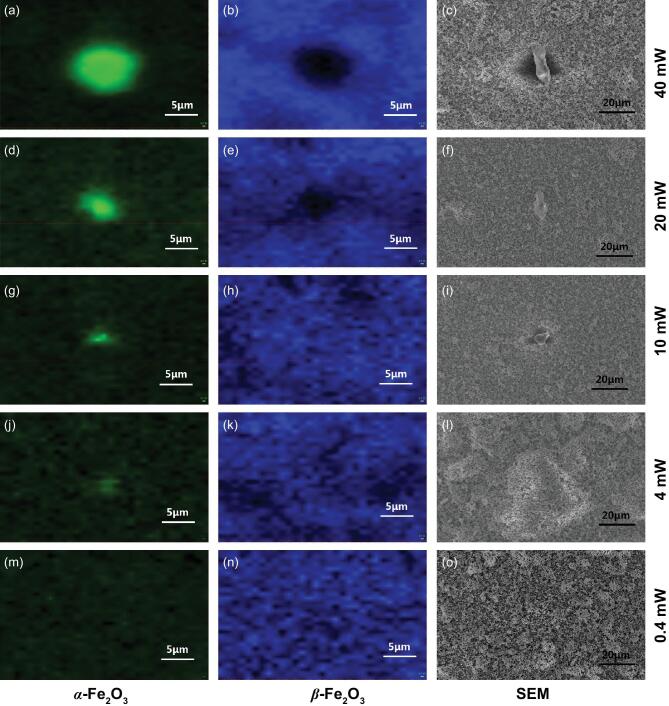
Raman mapping and SEM images of particle-assembled β-Fe_2_O_3_ films excited by a 0.4 mW laser of 785 nm after the irradiation of 785 nm laser with various power: (a-c) 40 mW, (d-f) 20 mW, (g-i) 10 mW, (j-l) 4 mW and (m-o) 0.4 mW. (a, d, g, j, m) α-Fe_2_O_3_; (b, e, h, k, n) β-Fe_2_O_3_.

Figure 4 shows Raman spectra mapping and SEM images of the particle-assembled β-Fe_2_O_3_ films without any necking treatment after laser illumination of 785 nm. When the β-Fe_2_O_3_ films were irradiated with 785 nm laser at 0.4 W power, no α-Fe_2_O_3_ signal appeared. With the increasing power of the 785 nm laser, the signals of α-Fe_2_O_3_ were enhanced, suggesting laser-induced phase transformation of β-Fe_2_O_3_ to α-Fe_2_O_3_. The SEM images of Fig. 4 also corroborate this result. The phase transformation of β-Fe_2_O_3_ to α-Fe_2_O_3_ is initiated by laser irradiation, likely as a result of the thermal effects of the laser. Therefore, low-power laser irradiation is required to avoid the phase transformation of metastable materials during Raman characterization to minimize its thermal effects. Thus, a 0.4 mW 785 nm laser is ideal for measuring the Raman spectra of β-Fe_2_O_3_.

**Figure 5.**
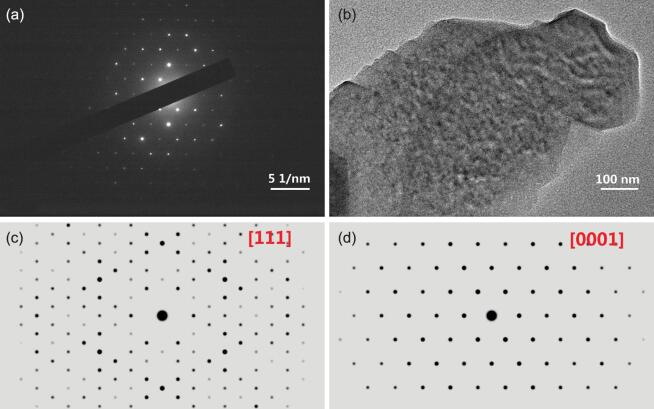
SAED and TEM images of the samples. (a) Measured SAED image of the β-Fe_2_O_3_ sample. (b) TEM image of the β-Fe_2_O_3_ sample. (c) Simulated SAED image of β-Fe_2_O_3_. (d) Simulated SAED image of α-Fe_2_O_3_.

Figure 5 shows transmission electron microscope (TEM) and selected area electron diffraction (SAED) images of the β-Fe_2_O_3_ samples. A simulated SAED image of α-Fe_2_O_3_ is shown in Fig. 5 for comparison. The measured SAED image of β-Fe_2_O_3_ samples (Fig. 5a) is in good agreement with the simulated SAED image of α-Fe_2_O_3_ (Fig. 5d) rather than the simulated SAED image of β-Fe_2_O_3_ samples (Fig. 5c), suggesting that the phase transformation of β-Fe_2_O_3_ to α-Fe_2_O_3_ induced by electron irradiation was also observed in this study. Note that the use of SAED to detect β-Fe_2_O_3_ should be done with caution. The phase transformation of β-Fe_2_O_3_ to α-Fe_2_O_3_ may be driven by laser irradiation, electron irradiation and heating (Supplementary Figs S3 and S4).

In our previous work, phase transformation of β-Fe_2_O_3_ to α-Fe_2_O_3_ occurred after annealing at 650°C [29]. Phase transformation of β-Fe_2_O_3_ was also proven by the appearance of the Raman peaks of α-Fe_2_O_3_ (Supplementary Fig. S3). In this study, Zr was introduced into the metastable β-Fe_2_O_3_ to inhibit phase transformation of β-Fe_2_O_3_ to α-Fe_2_O_3_. The formation energy of Zr_0.03_Fe_1.97_O_3_ (Zr-doped β-Fe_2_O_3_) was calculated as −0.16 eV relative to pure β-Fe_2_O_3_, suggesting that Zr can be doped into the lattice of β-Fe_2_O_3_ to form a more stable structure. The density of states (DOS) for Zr_0.03_Fe_1.97_O_3_ indicates that the band gap of β-Fe_2_O_3_ does not change obviously by doping Zr (Supplementary Fig. S5).

The XRD pattern of Zr-doped β-Fe_2_O_3_ (Supplementary Fig. S6) is in good agreement with β-Fe_2_O_3_ (JCPDS#39–0238), suggesting that there is no impurity phase after doping with Zr. The X-ray photoelectron spectroscopy in Supplementary Fig. S7 shows that Zr was doped into the sample. The absorption spectra of pure β-Fe_2_O_3_ and Zr-doped β-Fe_2_O_3_ are shown in Supplementary Fig. S8. After annealing, the band gap of pure β-Fe_2_O_3_ changed, whereas the band gap of Zr-doped β-Fe_2_O_3_ did not. In previous work, we have reported that the absorption spectrum of β-Fe_2_O_3_ varies after different calcining processes [35]. The change in band gap indicates that a phase transformation may occur. Indeed, after being annealed at 1023 K, the characteristic Raman peaks of α-Fe_2_O_3_ appeared in the Raman spectrum of the β-Fe_2_O_3_ sample (Supplementary Fig. S9a), indicating that there was a phase transformation. The Raman spectra of Zr-doped β-Fe_2_O_3_ did not change after being annealed at 1023 K (Supplementary Fig. S9b), indicating that there was no phase transformation. Note that the results of differential scanning calorimetry show that the phase transition temperature of Zr-doped β-Fe_2_O_3_ is clearly increased, as shown in Supplementary Fig. S10. The above results show that Zr doping effectively increases the phase transition temperature of metastable iron oxide.

**Figure 6.**
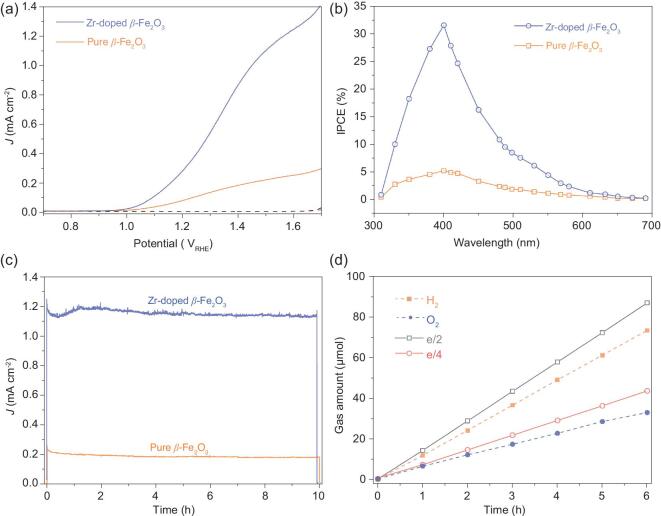
(a) Photocurrent density, (b) IPCE, (c) photochemical stability at 1.6 V_RHE_ and (d) Faradic efficiencies of the particle-assembled β-Fe_2_O_3_ films with Zr doping under illumination of standard simulated sunlight (100 mW cm^−2^) in 1 M NaOH electrolyte (pH = 13.6). Reference sample: α-Fe_2_O_3._

SEM images of the particle-assembled β-Fe_2_O_3_ photoanodes with and without Zr doping are shown in Supplementary Fig. S11. Figure 6a shows a comparison of the PEC performances of the particle-assembled β-Fe_2_O_3_ photoanodes with and without Zr doping under AM 1.5 G irradiation (100 mW cm^–2^).  The results show that the photocurrent of Zr-doped β-Fe_2_O_3_ photoanodes is much higher than that of pure β-Fe_2_O_3_ photoanodes. Zr-doped β-Fe_2_O_3_ photoanodes also exhibit much larger monochromatic incident photon-to-electron conversion efficiency (IPCE) than pure β-Fe_2_O_3_ photoanodes (Fig. 6b). Compared to the pure β-Fe_2_O_3_, the carrier density of Zr-doped β-Fe_2_O_3_ has been improved from 1.8 × 10^20^ to 4.6 × 10^20^ cm^−3^, which can be estimated from the Supplementary Fig. S12a. As shown in Supplementary Fig. S12b, the charge separation efficiency of Zr-doped β-Fe_2_O_3_ is improved by approximately four times at 1.6 V versus Reversible Hydrogen Electrode (RHE), compared to that of β-Fe_2_O_3_. The increase in the carrier concentration may favor the charge transport and contribute to the improvement in water splitting photocurrent of the Zr-doped β-Fe_2_O_3_ photoanode.

Under AM 1.5 G irradiation (100 mW cm^–2^), both Zr-doped and pure β-Fe_2_O_3_ photoanodes exhibit very good photochemical stability for water splitting during the PEC reaction over 10 h, as shown in Fig. 6c. In the case of particle-assembled Zr-doped β-Fe_2_O_3_ photoanodes, the Faradaic efficiencies for hydrogen production and oxygen production are 85% and 80%, respectively (Fig. 6d).  This result suggests that the photocurrent of the particle-assembled β-Fe_2_O_3_ photoanodes is mainly attributed to water splitting.

During the process of solar water splitting, β-Fe_2_O_3_ as a photoanode material may be exposed to standard simulated sunlight for a long time. To assess the possibility for phase transformation of β-Fe_2_O_3_ to α-Fe_2_O_3_ under simulated sunlight, the Raman spectra of the samples were measured before and after the photochemical stability testing. As shown in Supplementary Fig. S13, the phase of the β-Fe_2_O_3_ photoanodes before and after the reaction of PEC water splitting remains unchanged. In a typical PEC reaction, the intensity of the light used is not sufficient to promote the phase transformation of β-Fe_2_O_3_ particle-assembled films. Therefore, β-Fe_2_O_3_ is a promising photoanode for water splitting. The PEC performance of β-Fe_2_O_3_ may be improved further by not only the rational design of electrocatalysts but also the optimization of preparation methods [41,42].

## CONCLUSION

In conclusion, the Raman spectrum of β-Fe_2_O_3_ under excitation by a 785 nm laser with 0.4 W power shows 12 significant vibrational modes corresponding to β-Fe_2_O_3_. The phase transformation of metastable β-Fe_2_O_3_ to mature α-Fe_2_O_3_ may be observed under laser irradiation, electron irradiation and heating. Zr doping was introduced to particle-assembled β-Fe_2_O_3_ films, thus not only increasing the carrier concentration but also suppressing the phase transformation of β-Fe_2_O_3_. The PEC performance of the Zr-doped β-Fe_2_O_3_ photoanode was vastly boosted, in comparison with that of the pure β-Fe_2_O_3_ photoanode. This study demonstrates that metastable β-Fe_2_O_3_ remains stable during the PEC reaction and is a promising photoanode material for decomposing water, thus paving the road toward the use of β-Fe_2_O_3_ in solar water splitting (Supplementary Scheme S1).

## METHODS

In this study, β-Fe_2_O_3_ powder was synthesized by calcining the mixture of NaFe(SO_4_)_2_ (or 1.5% ZrSO_4_ doped NaFe(SO_4_)_2_) and NaCl in a muffle furnace at 450°C for 1 h [43]. As-prepared β-Fe_2_O_3_ powder was then deposited on fluorine-doped tin oxide (FTO) glass by electrophoretic deposition to prepare the particle-assembled films. In the case of necking treatment for the particle-assembled β-Fe_2_O_3_ films, 0.2 μmol of TiCl_4_ in methanol solution was dropped on the particle-assembled β-Fe_2_O_3_ films. Afterward, the films were annealed in a muffle furnace at 600°C (or 650°C) for 1 h.

Raman spectra of the samples were characterized with a confocal laser Raman spectrometer (Japan, Horiba, LabRAM Aramis, calibrated with silicon). Except for Fig. 1, all of the Raman spectra data acquisition used a 785 nm laser as the excitation source. Except for Fig. 3, all of the Raman spectra data acquisition used a 0.4 mW power laser as the excitation source. Raman imaging was obtained using a 785 nm, 0.4 mW laser. All laser-induced phase-change samples were confirmed using a 785 nm laser as the excitation source before laser irradiation to ensure that the samples contained no α-Fe_2_O_3_. The phosphor spectrum calculations were calculated in Material Studio with the Local Density Approximate, using the norm-conserving situation.

Density functional theory calculations on Zr-doped β-Fe_2_O_3_ were implemented in the VASP (Vienna Ab-initio Simulation Package) with a projected-augmented-wave method in the scheme of generalized-gradient approximation, whereas the strong on-site Coulomb repulsion among the localized Fe 3d electrons was described with the generalized-gradient approximation +U approach (‘U’ is ‘the strength of the on-site Coulomb interaction’).

SEM images were obtained on an SEM (Germany, Zessis, Ultra 55) and SAED images of the samples were obtained on a TEM (Japan, JEOL, Ltd. JEM2100).

PEC water splitting of the β-Fe_2_O_3_ particle-assembled films was carried out in 1 M NaOH solution (pH = 13.6) under AM 1.5 G standard simulated sunlight (American, Newport, Oriel Sol3A, 100 mW cm^−2^).

## Supplementary Material

nwaa039_Supplymentary_File
