# Supplementary material for: Paving the road toward the use of β-Fe2O3 in solar water splitting: Raman identification, phase transformation and strategies for phase stabilization
Source: Natl Sci Rev. 2020 Mar 9;7(6):1059–67. doi: 10.1093/nsr/nwaa039 (PMC8288852; doi:10.1093/nsr/nwaa039)
Supplement: nwaa039_Supplymentary_File [file nwaa039_supplymentary_file.docx]

Supplementary Information for

**Paving the Road toward the Use of β-Fe_2_O_3_ in Solar Water Splitting: Raman Identification, Phase Transformation, and Strategies for Phase Stabilization**

Ningsi Zhang^1^, Xin Wang^1^, Jianyong Feng^1^, Huiting Huang^1^, Yongsheng Guo^1^, Zhaosheng Li^1,2^* and Zhigang Zou^1,2^

^1^Collaborative Innovation Center of Advanced Microstructures, National Laboratory of Solid State Microstructures, College of Engineering and Applied Sciences, Nanjing University, 22 Hankou Road, Nanjing 210093, China, E-mail: [zsli@nju.edu.cn](mailto:zsli@nju.edu.cn)

^2^Jiangsu Key Laboratory of Nano Technology, Nanjing University, 22 Hankou Road, Nanjing 210093, China

**Supplementary Table S1.** Wyckoff positions of the atoms in the unit cell of β-Fe_2_O_3_. The irreducible representations of the Γ-point phonon modes.

| **Atom** | **Wyckoff notation** | **Irreducible representation** |
| --- | --- | --- |
| **Fe1** | 8b | A_u_+E_u_+3T_u_ |
| **Fe2** | 24d | A_g_+A_u_+E_g_+E_u_+5T_g_+5T_u_ |
| **O** | 48e | 3A_g_+3A_u_+3E_g_+3E_u_+9T_g_+9T_u_ |

**Supplementary Table S2.** Normal modes and selection rules for the Ia$\bar{3}$ structure.


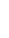


| **T_h_^7^** | **Vibrations** | **Selection rules** |
| --- | --- | --- |
| A_g_ | 4 | Raman |
| E_g_ | 4 | Raman |
| T_g_ | 14 | Raman |
| A_u_ | 5 | Inactive |
| E_u_ | 5 | Inactive |
| T_u_ | 17 | Infrared |

**Supplementary Table S3.** Previously published, experimentally observed (ω_expt_), and calculated (ω_calc_) Raman-active modes of β-Fe_2_O_3_.

| **Mode** | **Symmetry** | **Ref^#^** (cm^–1^) | **ω_expt_** (cm^–1^) | **ω_calc_** (cm^–1^) | **Δω** (cm^–1^) |
| --- | --- | --- | --- | --- | --- |
| M1 | T_g_ | 158 | 158 | 179 | -21 |
| M2 | T_g_ | 168 | 169 | 196 | -27 |
| M3 | T_g_ | 208 |  | 224 |  |
| M4 | A_g_ | 235 | 234* | 229 | 5 |
| M5 | E_g_ |  | 258* | 254 | 4 |
| M6 | T_g_ | 273 | 274 | 266 | 8 |
| M7 | T_g_ | 312 | 314 | 325 | -11 |
| M8 | T_g_ | 326 | 328 | 336 | -8 |
| M9 | T_g_ | 367 | 368 | 365 | 3 |
| M10 | E_g_ | 383 | 386 | 383 | 3 |
| M11 | T_g_ |  | 386 | 389 | -3 |
| M12 | A_g_ |  | 397 | 393 | 4 |
| M13 | T_g_ |  |  | 421 |  |
| M14 | E_g_ |  |  | 461 |  |
| M15 | T_g_ |  |  | 470 |  |
| M16 | T_g_ |  | 522 | 518 | 4 |
| M17 | T_g_ |  |  | 539 |  |
| M18 | T_g_ |  |  | 548 |  |
| M19 | A_g_ |  |  | 581 |  |
| M20 | A_g_ |  |  | 597 |  |
| M21 | T_g_ |  |  | 625 |  |
| M22 | E_g_ |  | 635 | 633 | 2 |

# Y. Q. [Liang, R.](http://apps.webofknowledge.com/OneClickSearch.do?product=UA&search_mode=OneClickSearch&SID=8FXk8Kn6LavYYd37wtZ&field=AU&value=Liang,%20YQ&ut=50476210&pos=1&excludeEventConfig=ExcludeIfFromFullRecPage) [van de Krol,](http://apps.webofknowledge.com/OneClickSearch.do?product=UA&search_mode=OneClickSearch&SID=8FXk8Kn6LavYYd37wtZ&field=AU&value=van%20de%20Krol,%20R&ut=90154960&pos=2&excludeEventConfig=ExcludeIfFromFullRecPage) Influence of Si dopant and SnO_2_ interfacial layer on the structure of the spray-deposited Fe_2_O_3_ Films. *Chem. Phys. Lett*. **479**, 86(2009).**Supplementary Table S4.** Experimentally observed (ω_expt_) and calculated (ω_calc_) infrared-active modes of β-Fe_2_O_3_.

| **Mode** | **Symmetry** | **ω_expt_** (cm^–1^) | **ω_calc_** (cm^–1^) | **Δω** (cm^–1^) |
| --- | --- | --- | --- | --- |
| M0 | T_u_ |  | -0.026 |  |
| M1 | T_u_ |  | 167 |  |
| M2 | T_u_ |  | 179 |  |
| M3 | T_u_ |  | 230 |  |
| M4 | T_u_ |  | 248 |  |
| M5 | T_u_ |  | 264 |  |
| M6 | T_u_ |  | 294 |  |
| M7 | T_u_ |  | 330 |  |
| M8 | T_u_ |  | 344 |  |
| M9 | T_u_ |  | 390 |  |
| M10 | T_u_ | 419 | 413 | 5 |
| M11 | T_u_ | 439 | 450 | -11 |
| M12 | T_u_ | 457 | 480 | -23 |
| M13 | T_u_ | 546 | 526 | -2 |
| M14 | T_u_ |  | 550 |  |
| M15 | T_u_ | 577 | 563 | 14 |
| M16 | T_u_ | 634 | 605 | 29 |

The phosphor spectrum calculations were calculated in Material Studio with the Local Density Approximate, using the norm-conserving situation. M0 corresponds to a virtual Phonon.


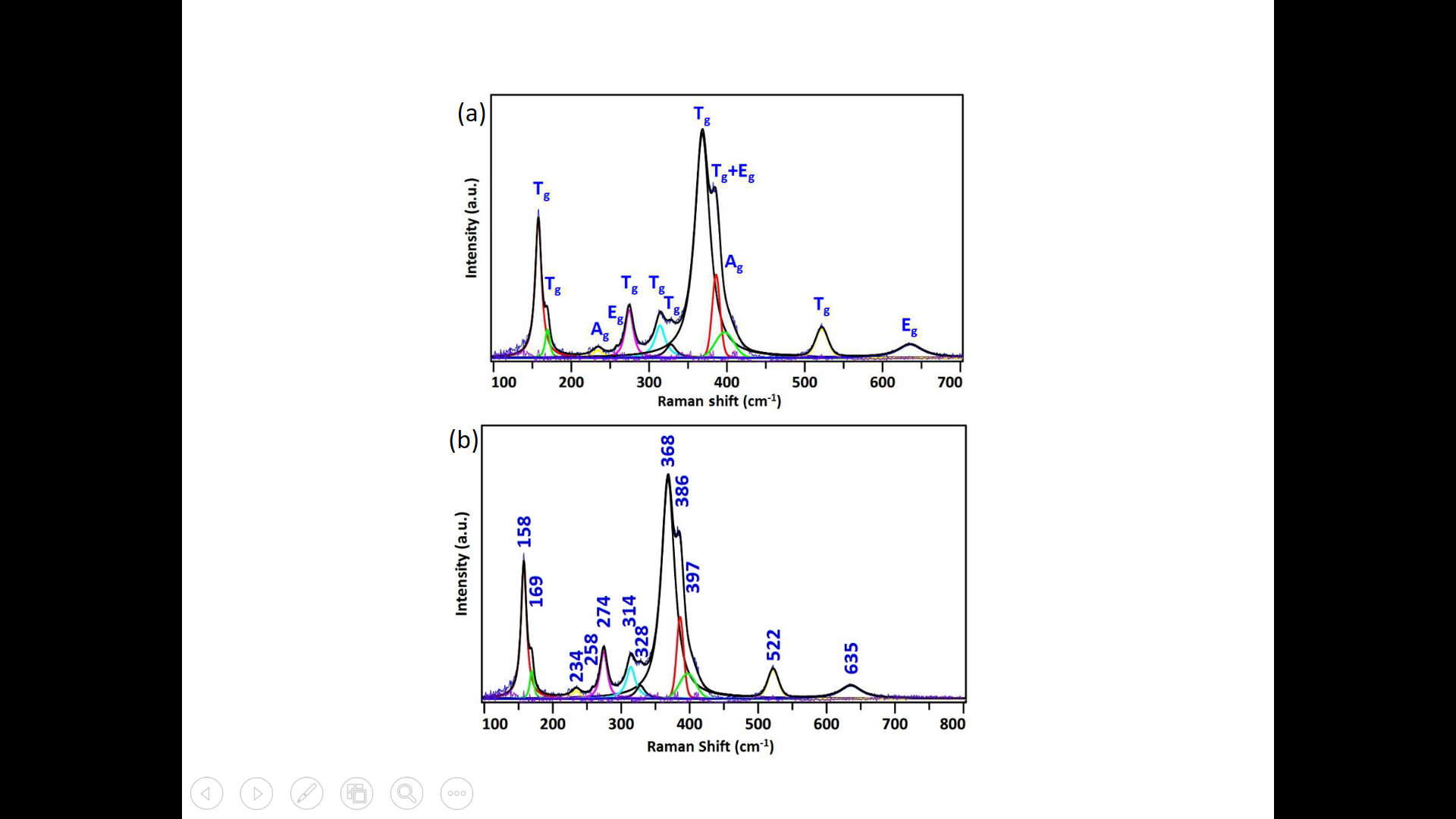


**Supplementary Figure S1.** Raman spectra and vibration modes of β-Fe_2_O_3_ using a 785 nm laser of 0.4 W.


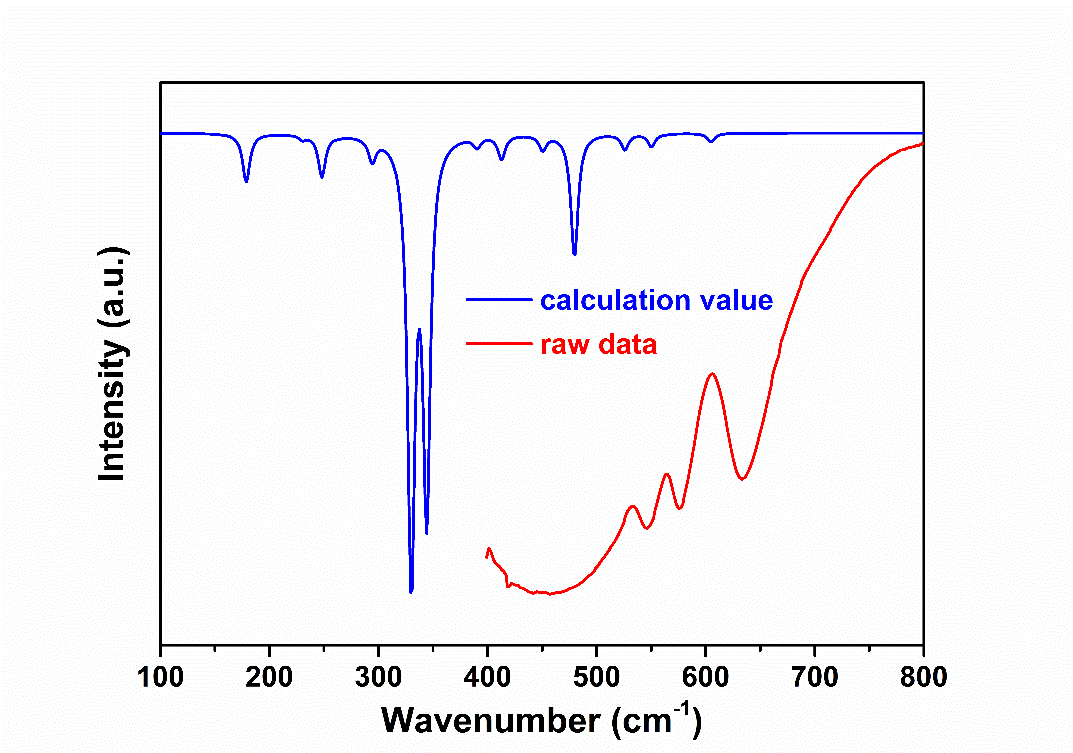


**Supplementary Figure S2.** Experimental infrared absorption spectrum and theoretical calculation results of β-Fe_2_O_3_.


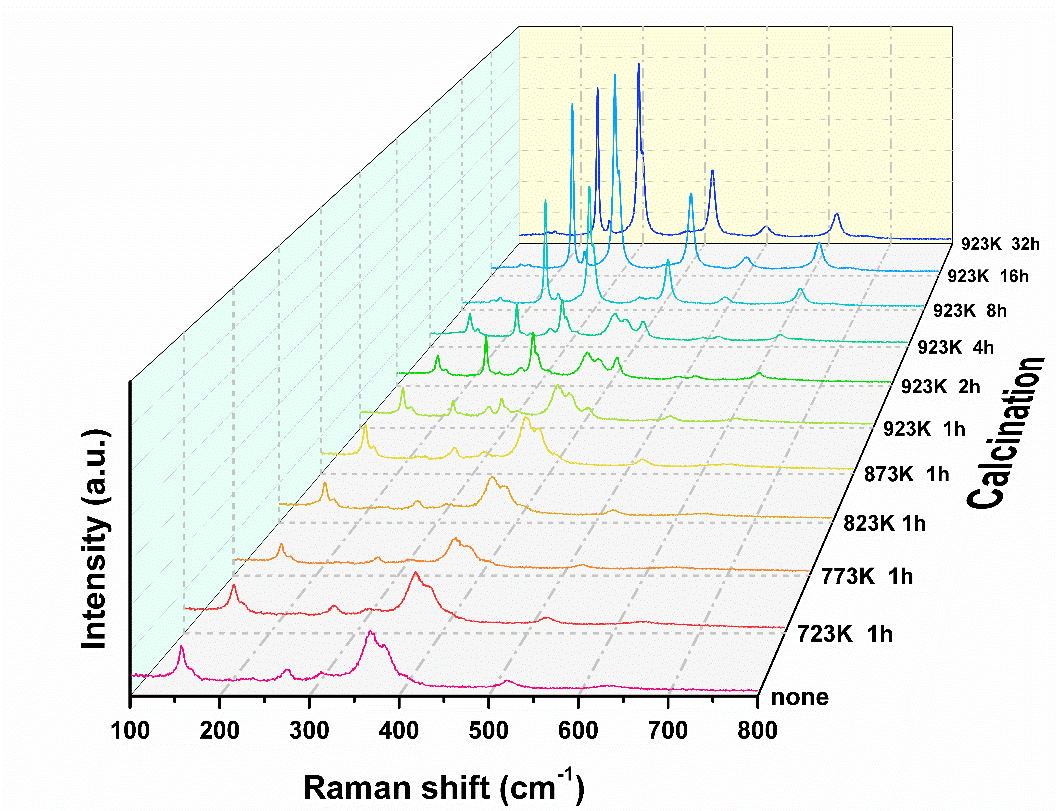


**Supplementary Figure S3.** Raman spectra of β-Fe_2_O_3_ photoanodes as a function of different annealing processes using a 785 nm laser of 0.4 W.


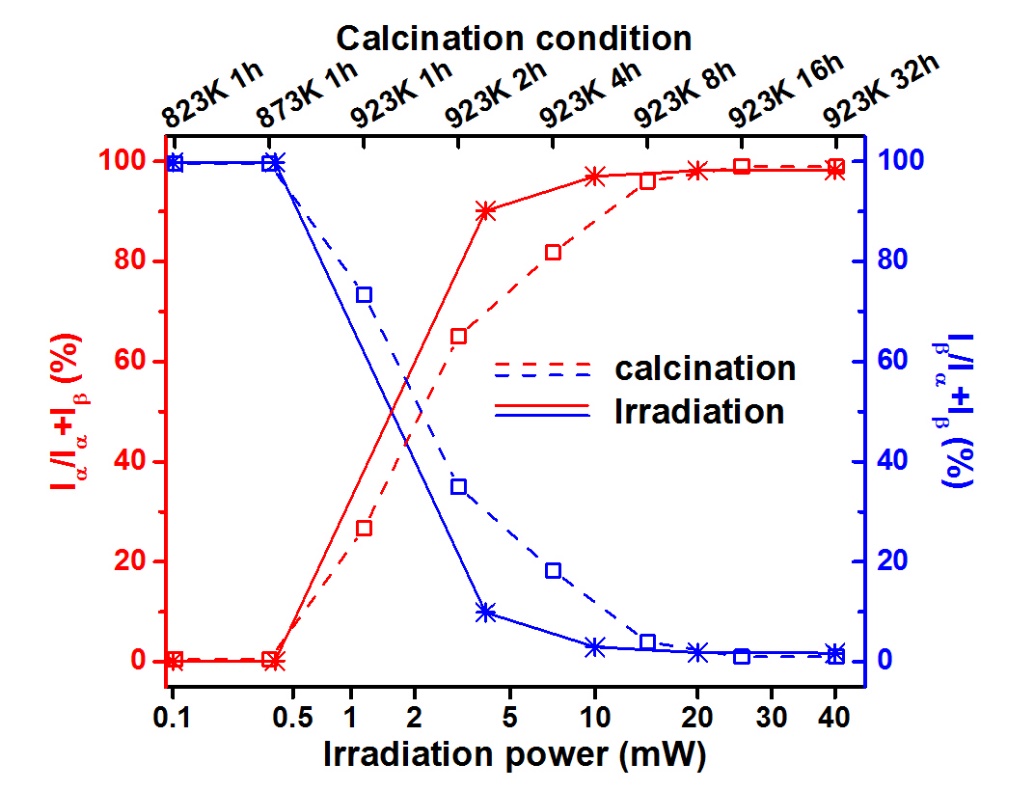


**Supplementary Figure S4.** Relative Raman intensity of β-Fe_2_O_3_ photoanodes as a function of different annealing processes and 785 nm laser irradiation power.

**Supplementary Figure S5.** Density of states (DOS) for Zr-doped β-Fe_2_O_3_ (Zr_0.03_Fe_1.97_O_3_) and pure β-Fe_2_O_3_.

Density functional calculations in this study were implemented by the Vienna ab initio simulation package (VASP) with projected-augmented-wave (PAW) method. The Generalized-gradient approximation (GGA) was adopted for the exchange-correlation functional. The strong on-site Coulomb repulsion among the localized Fe 3d electrons is described by using the GGA +U approach. The cut-off energy is 500 eV, which has been sufficiently examined for computational accuracy. The formation energy of Zr impurity is calculated as following:

$\Delta E=E_{Zr:{Fe}_{2}O_{3}}^{t}-E_{{Fe}_{2}O_{3}}^{t}-\mu_{Zr}{+\mu}_{Fe}$ (1)

where $E_{Zr:{Fe}_{2}O_{3}}^{t}$ is formation energy of β-Fe_2_O_3_ supercell with Zr dopants, the $E_{{Fe}_{2}O_{3}}^{t}$is formation energy of pure β-Fe_2_O_3_ supercell, $\mu_{Zr}$ is the chemical potential of element Zr and it is approximated to the formation energy of elemental Zr, $\mu_{Fe}$ is approximated to the formation energy of elemental Fe.


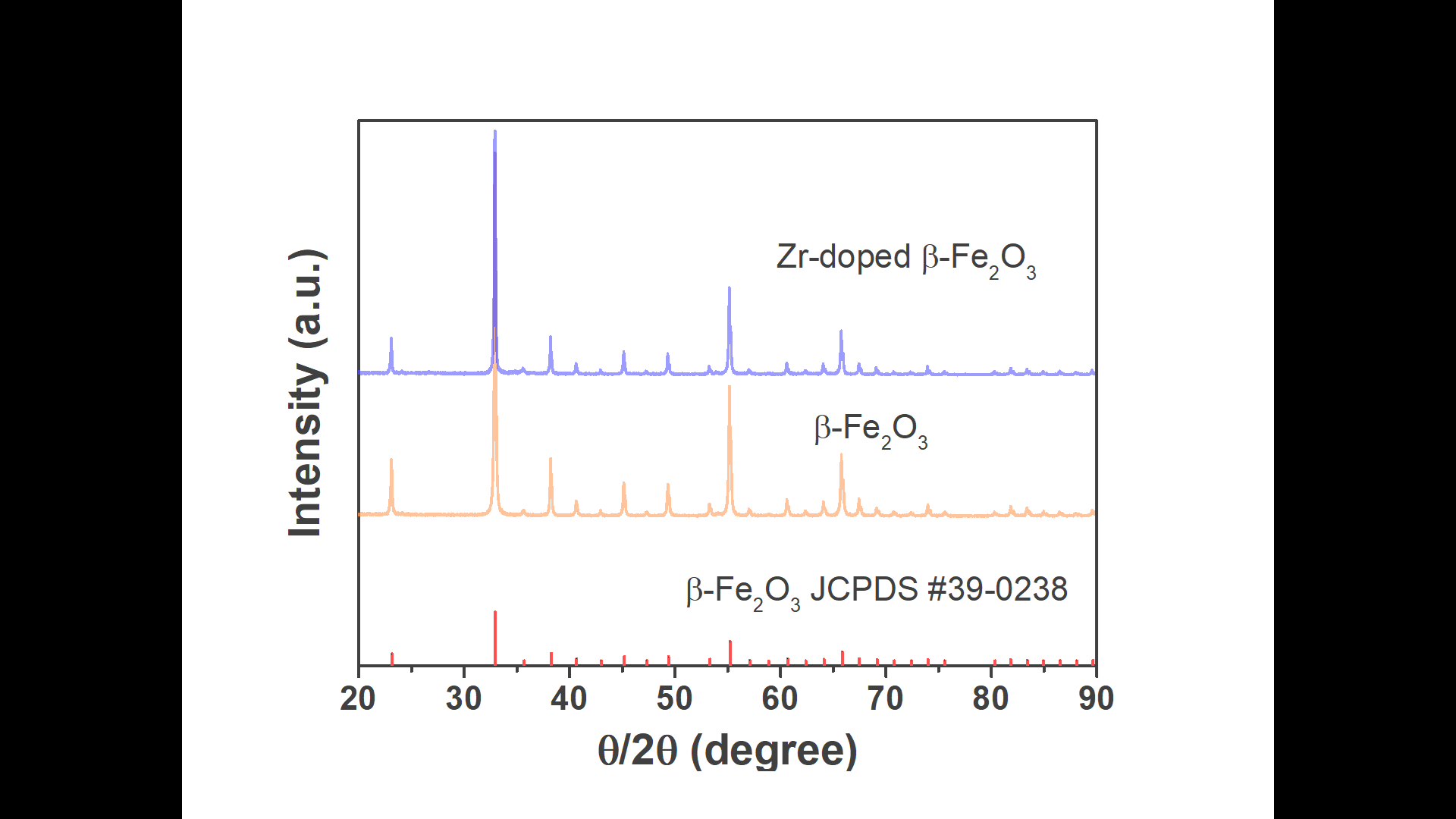


**Supplementary Figure S6.** XRD patterns of β-Fe_2_O_3_ with and without Zr doping.


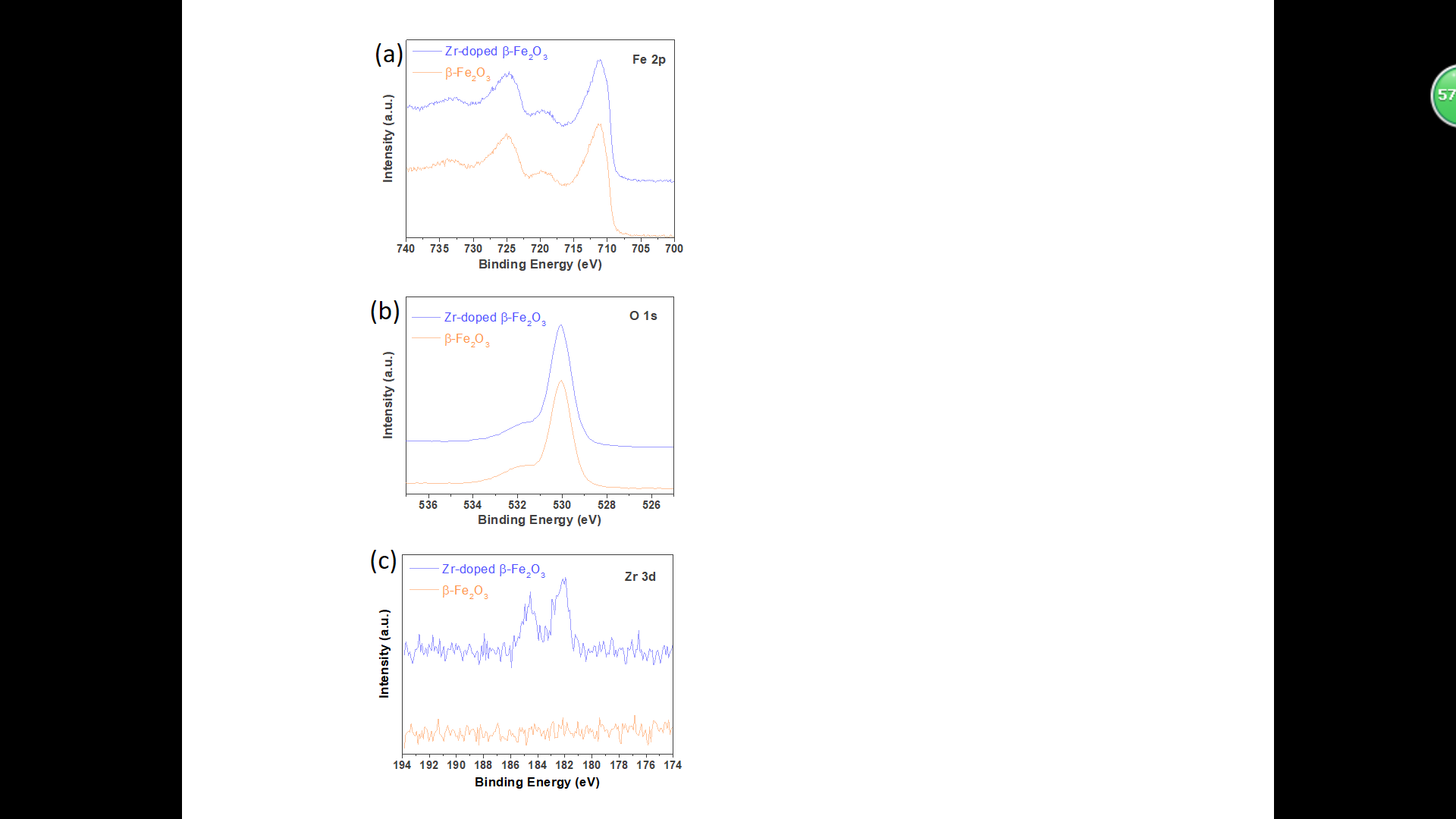


**Supplementary Figure S7.** XPS spectra of β-Fe_2_O_3_ with and without Zr doping.


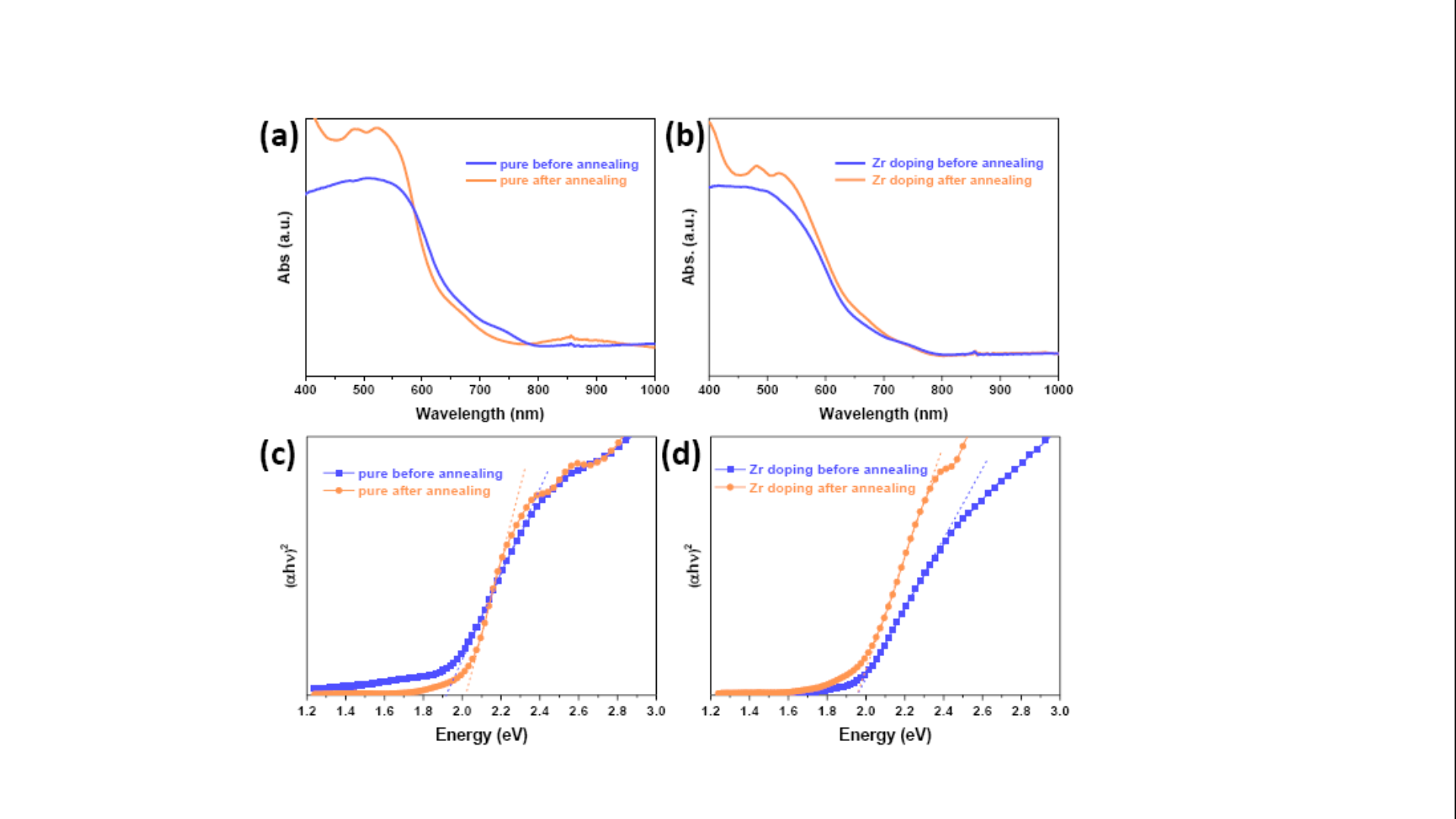


**Supplementary Figure S8.** Absorption spectra and optical band gap of (a), (c) pure β-Fe_2_O_3_ and (b), (d) Zr-doped β-Fe_2_O_3_ before and after annealing at 923 K for 1 h and 1023 K for 5 min.


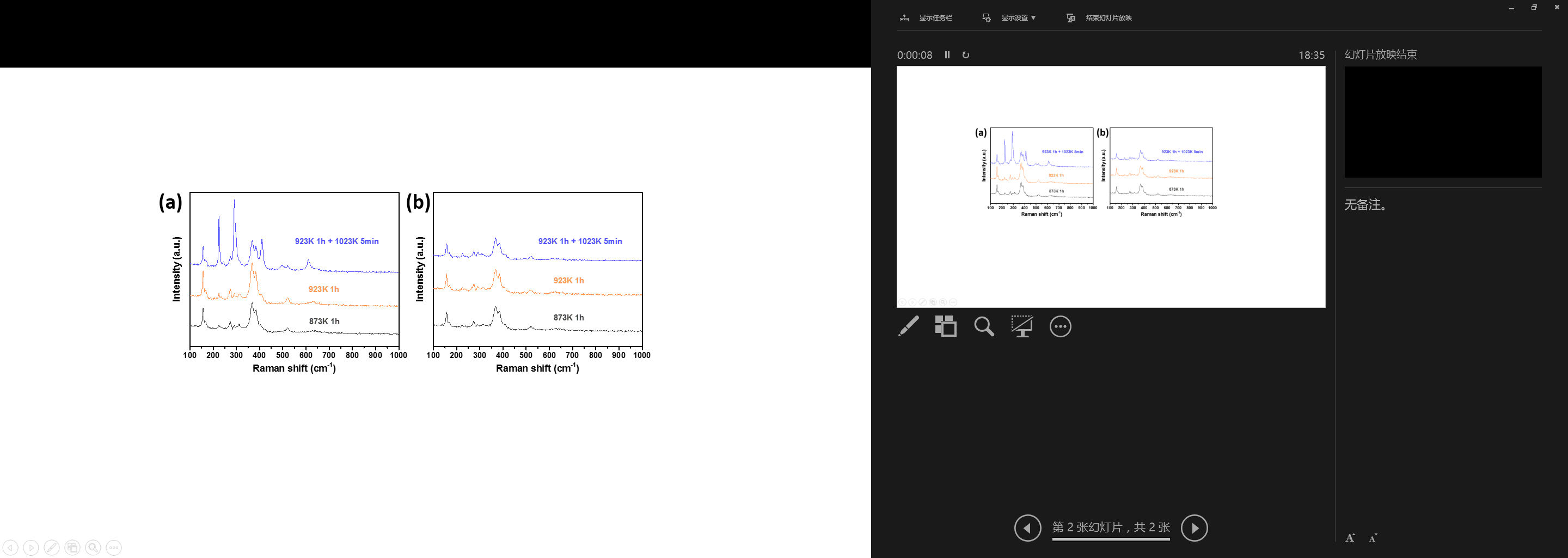


**Supplementary Figure S9.** Raman spectra of (a) pure and (b) Zr-doped β-Fe_2_O_3_ photoanode films as a function of different annealing temperatures using a 785 nm laser of 0.4 W.


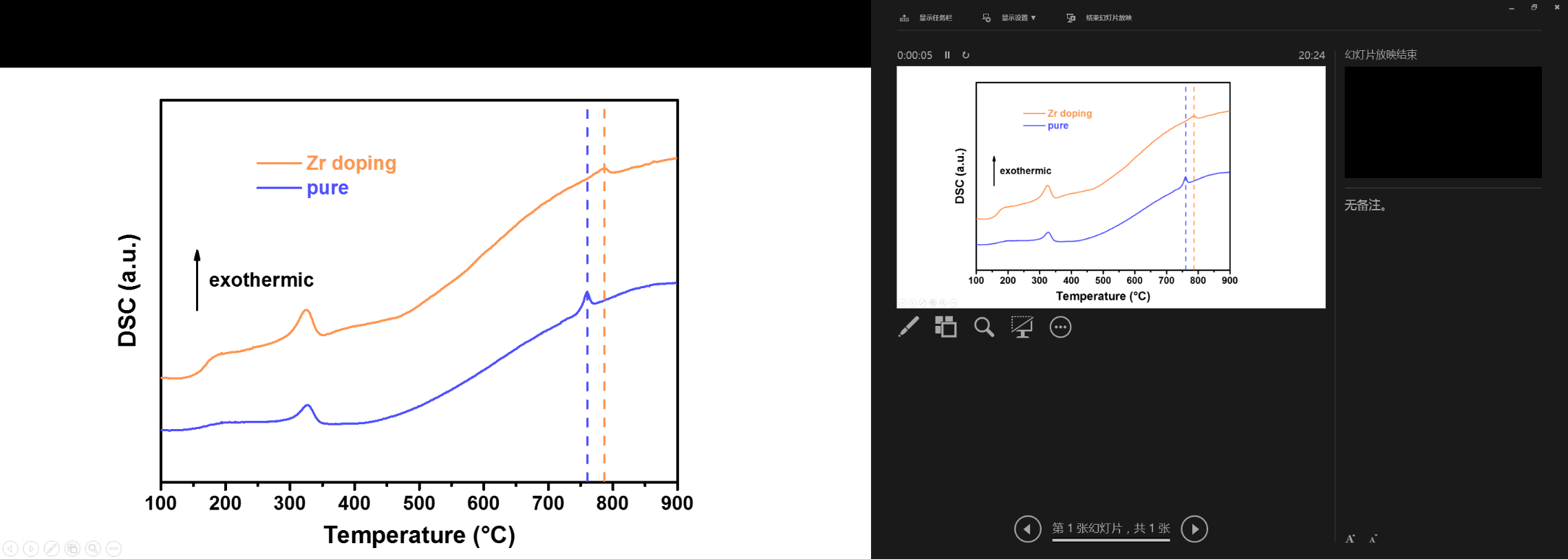


**Supplementary Figure S10.** Differential scanning calorimetry (DSC) of β-Fe_2_O_3_ with and without Zr doping.


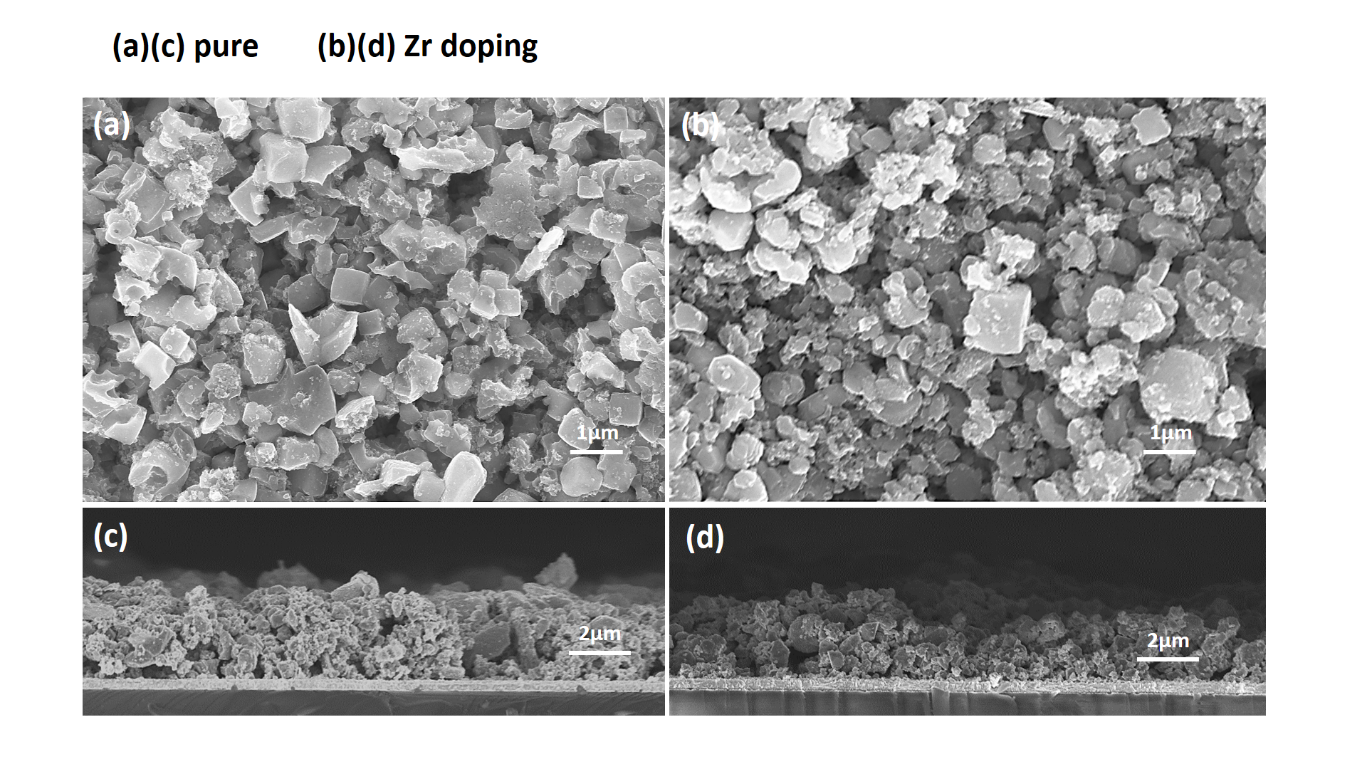


**Supplementary Figure S11.** SEM images of β-Fe_2_O_3_ photoanode films (a, c) without and (b, d) with Zr doping.


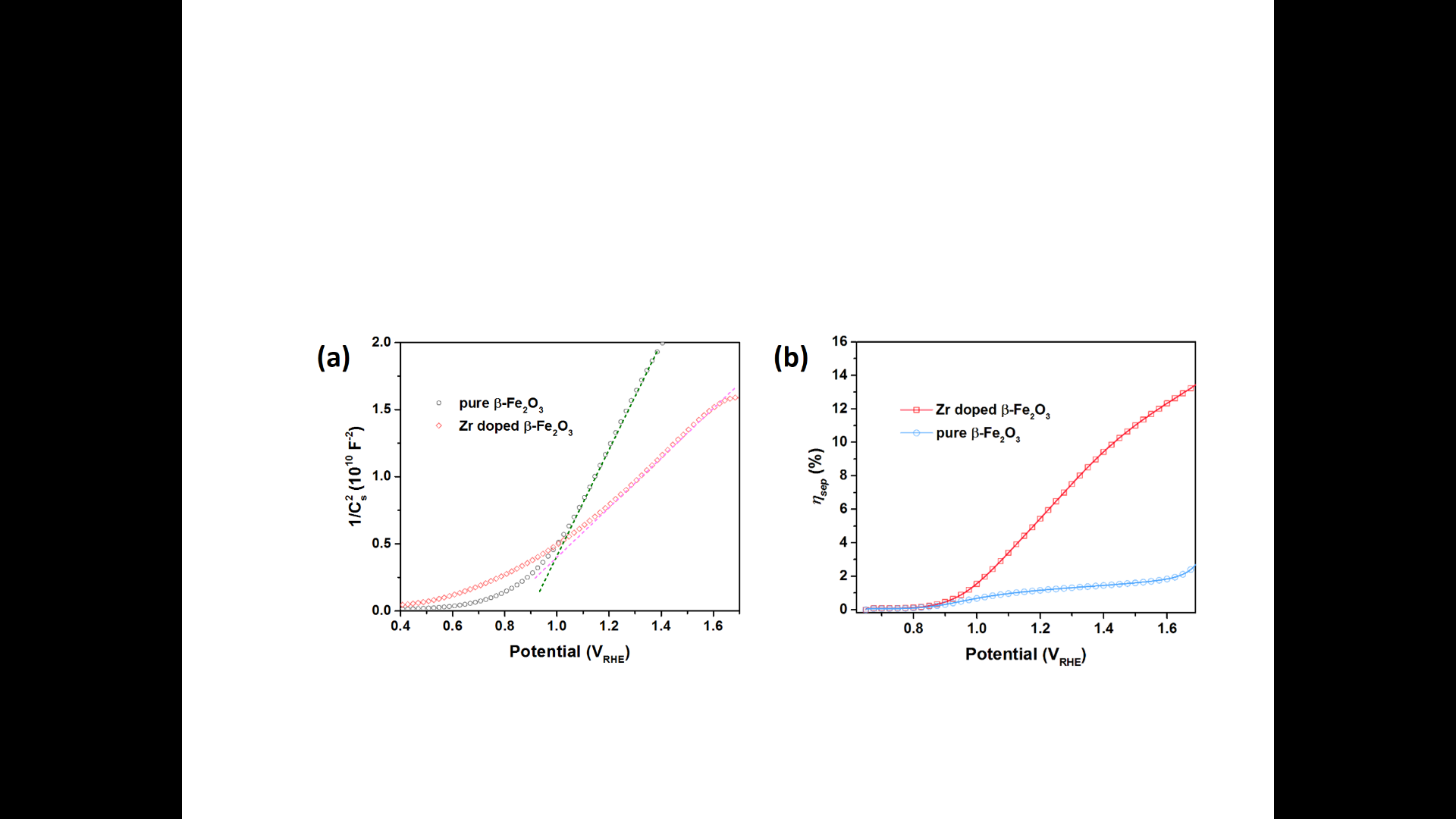


**Supplementary Figure S12.** (a) Mott-schottky curves and (b) charge separation efficiencies of β-Fe_2_O_3_ photoanodes.

**Supplementary Figure S13.** Raman spectra of the β-Fe_2_O_3_ photoanode (using a 785 nm laser of 0.4 W) before and after 10 h PEC testing under illumination of standard simulated sunlight (Newport, Oriel Sol3A, AM 1.5 G, 100 mW cm^-2^) at 1.6 V_RHE_ in a 1 M NaOH electrolyte (pH=13.6).


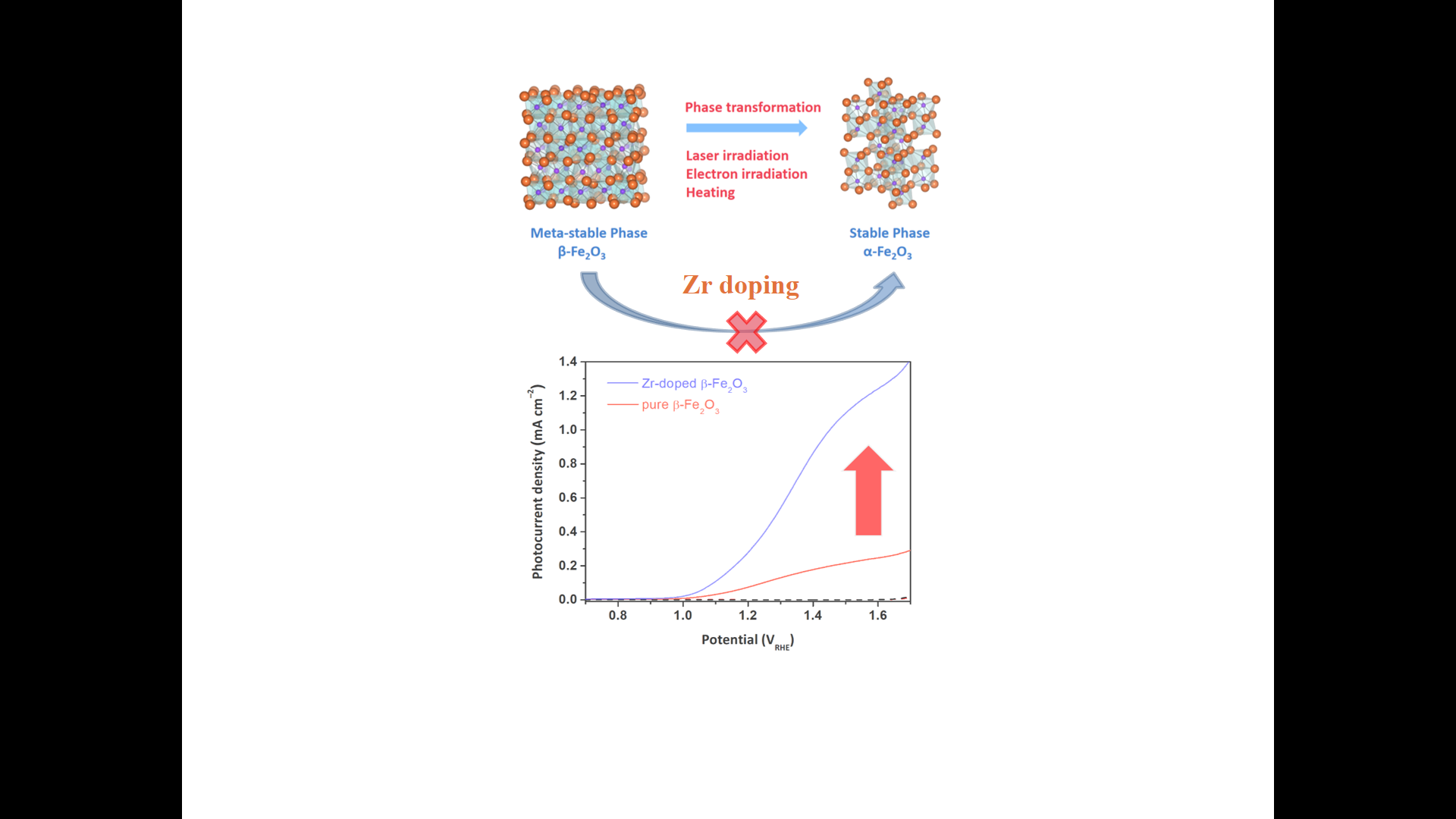


**Supplementary Scheme S1.** Improved PEC performance of metastable β-Fe_2_O_3_ photoanode by hindering the phase transformation.
